# Factors associated with recovery from 1 minute Apgar score <4 in live, singleton, term births: an analysis of Malaysian National Obstetrics Registry data 2010–2012

**DOI:** 10.1186/s12884-017-1293-9

**Published:** 2017-04-08

**Authors:** Ravichandran Jeganathan, Shamala D. Karalasingam, Julia Hussein, Pascale Allotey, Daniel D. Reidpath

**Affiliations:** 1 Department of Obstetrics and Gynaecology, Sultanah Aminah Hospital, Ministry of Health Malaysia, Johor Bahru, Malaysia; 2 National Obstetric Registry, Clinical Research Centre, Ministry of Health Malaysia, Kualar Lumpur, Malaysia; 3 Immpact, University of Aberdeen, Aberdeen, Scotland UK; 4 South East Asia Community Observatory (SEACO), Monash University Malaysia, Bandar Sunway, Malaysia; 5 Jeffrey Cheah School of Medicine and Health Sciences, Monash University Malaysia, Bandar Sunway, Selangor 46150 Malaysia

**Keywords:** Apgar score, Newborn, Term birth, Registries, Malaysia

## Abstract

**Background:**

The neonatal Apgar score at 5 min has been found to be a better predictor of outcomes than the Apgar score at 1 min. A baby, however, must pass through the first minute of life to reach the fifth. There has been no research looking at predictors of recovery (Apgar scores ≥7) by 5 min in neonates with 1 min Apgar scores <4.

**Methods:**

An analysis of observational data was conducted using live, singleton, term births recorded in the Malaysian National Obstetrics Registry between 2010 and 2012. A total of 272,472 live, singleton, term births without congential anomalies were recorded, of which 1,580 (0.59%) had 1 min Apgar scores <4. Descriptive methods and bi- and multi-variable logistic regression were used to identify risk factors associated with recovery (5 min Apgar score ≥7) from 1 min Apgar scores <4.

**Results:**

Less than 1% of births have a 1 min Apgar scores <4. Only 29.4% of neonates with 1 min Apgar scores <4 recover to a 5 min Apgar score ≥7. Among uncomplicated vaginal deliveries, after controlling for other factors, deliveries by a doctor of neonates with a 1 min Apgar score <4 had odds of recovery 2.4 times greater than deliveries of neonates with a 1 min Apgar score <4 by a nurse-midwife. Among deliveries of neonates with a 1 min Apgar score <4 by doctors, after controlling for other factors, planned and unplanned CS was associated with better odds of recovery than uncomplicated vaginal deliveries. Recovery was also associated with maternal obesity, and there was some ethnic variation – in the adjusted analysis indigenous (Orang Asal) Malaysians had lower odds of recovery.

**Conclusions:**

A 1 min Apgar score <4 is relatively rare, and less than a third recover by five minutes. In those newborns the qualification of the person performing the delivery and the type of delivery are independent predictors of recovery as is maternal BMI and ethnicity. These are associations only, not necessarily causes, and they point to potential areas of research into health systems factors in the labour room, as well as possible biological and cultural factors.

## Background

The most consistently used measure of neonatal health in the few minutes after delivery is the Apgar score, [1–3] providing labor ward staff with a shared understanding of a newborn’s status, and the possible need for and response to resuscitation [4–6]. The assessment is usually made a number of times within the first ten minutes of birth, usually at 1, 5, and 10 min.

Research has generally focused on the Apgar score at 5 min, and more specifically the relationship between the Apgar score at 5 min and future neonatal and infant outcomes [7–10]. There appears to have been little or no research examining the relationship between the Apgar score at 1 min and the Apgar score at 5 min. While the Apgar score at 5 min is a better predictor of later outcomes than the Apgar score at 1 min, [10] there is a necessary temporal process involved, and a neonate must pass through the first minute of life to reach the fifth. Understanding the factors associated with the transition from intrauterine to extrauterine life, particularly for neonates with 1 min Apgar scores <4, has the potential to improve care.

The major difference between the Apgar assessment at 1 and 5 min is that the Apgar assessment within the first minute of birth provides an indication of intrapartum health and the neonatal response to the “trauma” of birth [6]. A 1 min Apgar score <4 is likely to trigger a range of protocols which may (or may not) lead to resuscitative intervention. The Apgar assessment at 5 min provides an indication of a neonate’s sustained capacity to survive and thrive. It is for this reason that the second Apgar assessment, made at 5 min, is a better predictor of later outcomes than the Apgar score at 1 min (7).

For a neonate with an initially poor Apgar score, the difference between the 1st and 5th minute Apgar scores indicates the capacity to recover and the potential need for ongoing management. Variation in recovery rates between the first and fifth minute provides significant clinical information and may, furthermore, provide insight into health systems issues in the intrapartum window. Understanding the factors associated with the transition between the scores, particularly for neonates with 1 min Apgar scores <4 has the potential to bring into relief points for intervention or further investigation. With a focus on term, newborns with low one minute Apgar scores, we examine:

- The patterns of Apgar score change in newborns from 1 to 5 min; and
- The factors associated with recovery from a 1 min Apgar score <4;

The study draws on data from the Malaysian, National Obstetrics Registry (NOR).

## Methods

### The National Obstetrics Registry

Malaysia is a multi-ethnic country with a population around 29.5 million, comprising a majority Malay population with significant Chinese, Indian, and indigenous (Orang Asal [11]) minorities. The estimated total fertility rate for the population is 2.1 [12]. The health system is a mixed public-private system, with approximately 85% of all deliveries occurring in government facilities. In the public system antenatal care is generally nurse-midwife led; data are not readily available from the private system.

The Malaysian National Obstetrics Registry (NOR) was established in July 2009 and has become one of the largest active birth registries in the world adding about 100,000 births per annum. Routinely collected data from 2010 to 2012 were used from the 14 tertiary hospitals covered by the NOR—one from each of the 13 States and the Federal Territory of Kuala Lumpur. The hospitals are federally funded and operate according to common policies. The labour rooms in all the hospitals are staffed by appropriately qualified medical and nursing staff who are trained in neonatal resuscitation techniques.

The Ministry of Health estimated 491,365 total live births in Malaysia in 2011, and the NOR registered approximately 27.3% of these. A complete description of the NOR can be found in the annual reports, [13, 14] and the website [http://www.acrm.org.my/nor/]. Ethical approval for the research was provided by the Medical Research and Ethics Committee of the Ministry of Health, Malaysia (Approval number: NMRR15-620-25530).

### Sample

**Fig. 1:**
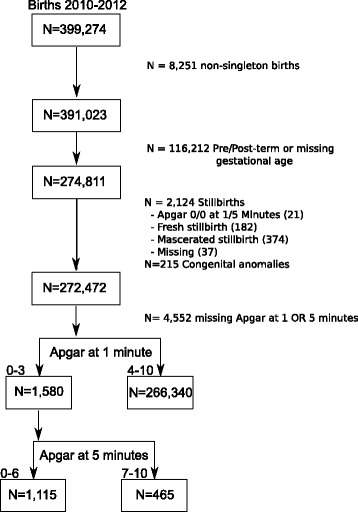
The data flow in the current study

A total of 399,274 births were registered between 1 January 2010 and 31 December 2012, of which 272,472 were live, term, singleton births, with complete 1 and 5 min Apgar scores. Births directly recorded as stillbirths (*n* = 593) or births with both 1 and 5 min Apgar scores of zero (*n* = 21) were excluded from the analysis. Neonates with congenital anomalies (*n* = 215) were also excluded from the analysis. The focus of the study was 1,580 singleton, term, deliveries (848 male, 582 female, and 140 missing) in which the neonate had a 1 min Apgar score <4. As a point of initial contrast, the full cohort (*n* = 272,472) over the range of Apgar scores (0–10) was retained. The sampling frame is shown in Fig. 1.

### Measures

The outcome measure of interest was a binary variable indicating Apgar recovery at 5 min. Apgar scores were categorised as <4, 4–6 and ≥7 [10, 15]. If a neonate had an Apgar score <4 and subsequently received a 5 min Apgar score ≥7, they were categorised as recovered, if their subsequent 5 min Apgar score was <7, they were categorised as not recovered. The Apgar score is recorded by the nurse-midwife in the labour room according to their clinical assessment. All term births with no congenital anomalies with an Apgar score of 0 (no respiration and no pulse) would be subject to resuscitation.

The predictors used in the study were drawn from the limited socio-demographic factors available in the NOR, maternal medical history, obstetric history, and the actual delivery. The final categories for some factors were selected following a preliminary examination of the data to take account of small cell sizes, and similar outcomes in adjacent ordered or related categories. Birthweight for instance was categorised as “Low”, “Not Low” and “Missing”. Eight neonates (4 recovered, 4 not recovered) with implausibly large birthweights were recoded as having “Missing” birthweights. Where possible, missing data were retained as a separate category to avoid sample loss, and to allow the explicit modelling of the missing data. In one case, the single record with missing age data for the mother was allocated to the most common age category (20–29). The predictors and their associated categories are shown below with base categories underlined:

- Age: <20; *20*–*29*; 30–39; 40+
- Race/Ethicity: *Malay*, Chinese, Indian, Orang Asal (indigenous), Other (including migrants), and Missing. The recorded category is based on details recorded on the mother’s National Registration Identity Card.
- BMI: Obese; *non-Obese*; Missing. The BMI classification is based on the WHO Asian cut-off of 27.5, [16] where the mothers height and weight are recorded at the first antenatal visit, usually in the first trimester.
- Diabetes: *No diabetes*; Diabetes (combining gestational diabetes or a pre-existing diabetes).
- Parity: *1*; 2–3; 4–5; 6+. It should be noted that no mother in this study can be P0, by definition.
- Maternal haemoglobin at delivery: ≤11; *>11*
- Neonate sex: *Female*; Male; Missing
- Birth weight: Low (<2500 g); *Not Low* (≥2500 g); Missing.
- Foetal distress (based on clinical judgement): Present; *Absent*
- Delivery type: Uncomplicated *vaginal delivery* (Vaginal); Breech vaginal delivery (Breech); Vacuum Extraction; Elective Caesarean section (Elective CS); Emergency Caesarean section (Emerg CS); Missing. The standing procedures in the 14 state tertiary hospitals is for spinal anaesthesia to be used for CS.
- Delivered by: *Medical Doctor*; Nurse-midwife; Other. Clinical staff working in the labor rooms are trained in neonatal resuscitative techniques.

A number of factors initially considered were later excluded because of small cell sizes including the presence or absence of heart disease, hypertension, and a maternal blood disorder. Socioeconomic measures including maternal education and household income were excluded because data were not consistently collected across the three years of the study.

### Data analysis

The data analysis was conducted in three stages. In the first stage a descriptive analysis of the relationship between 1 and 5 min Apgar was conducted; and the probability of having a 5 min Apgar ≥7 given each possible 1 min Apgar score was estimated with 95% confidence intervals. Thus, for neonates within each of the 11 possible 1-min Apgar scores categories (0–10), the probability of them having a 5-min Apgar score ≥7 was estimated as the conditional proportion. In the second stage, a series of bivariable logistic regression models of Apgar recovery were estimated for those with 1 min Apgar scores <4 using each predictor in turn. Odds ratios were estimated with appropriate 95% confidence intervals. In the third stage multivariable logistic regression models were developed. It was determined a priori that all variables from the bivariable analyses would be included in the multivariable analyses. However, two separate models were developed to overcome collinearity between the delivery type (vaginal, emergency Caesarean, etc.,) and the personnel involved in the delivery (doctor, nurse-midwife, etc.). The outcome in both cases was Apgar recovery; and odds ratios were estimated with appropriate 95% confidence intervals. The first model was restricted to uncomplicated vaginal deliveries by doctors and nurse-midwives and included all the remaining factors from the bivariable analyses. The second analysis was restricted to deliveries by Doctors, involving all delivery types, and all the other factors from the bivariable analyses.

Logistic regression modeling used a Generalized Linear Modeling (GLM) approach with a binomial link function and 95% confidence intervals were based on the profiled log-likelihood functions. Analyses were conducted in the R statistical environment [17].

## Results

Around 97% of all the term, singleton births achieved a 1 min Apgar score ≥7, and of those the vast majority (99.94%) maintained a 5 min Apgar score ≥7. Approximately 0.59% (1,580/267,920) of births had a 1 min Apgar scores <4.

The cross tabulation of 1 and 5 min Apgar scores for the 267,920, live, singleton, term births is shown in Table 1 (a scatter-plot of the data can also be found here [18]). The neonates born with 1 min Apgar scores <4 are in cells highlighted in grey—the focus of later analyses. The most striking feature of the table is the preponderance of neonates appearing in the cells in the lower right corner, indicating that neonates with 1 min Apgar scores ≥7 are generally distributed into even better 5 min Apgar scores.

**Table 1:** The 1 minute × 5 minute Apgar scores in 267,920 term, singleton, live births

	5 minute Apgar score											
1 minute Apgar Score	0	1	2	3	4	5	6	7	8	9	10	Total
0	0	4	2	3	0	0	1	1	0	3	5	19
1	219	44	19	19	28	30	18	12	9	9	17	424
2	1	179	31	16	46	64	57	32	39	30	10	505
3	1	2	100	22	35	72	102	78	79	93	48	632
4	1	2	4	45	50	76	108	162	182	186	98	914
5	4	2	0	0	18	39	113	295	429	559	245	1,704
6	0	2	1	0	2	8	41	315	920	1,355	473	3,117
7	1	3	0	0	0	6	10	46	1,404	3,407	1,084	5,961
8	0	7	0	0	1	8	7	11	275	42,765	3,517	46,591
9	3	85	1	2	2	7	9	10	30	78,235	129,652	208,036
10	0	0	0	0	0	0	0	0	0	4	13	17
Total	230	330	158	107	182	310	466	962	3,367	126,646	135,162	267,920

Of the relatively few who did not achieve a 1 min Apgar score ≥7 (7,315/267,920), 77.6% achieved a 5 min Apgar ≥7.

**Fig. 2:**
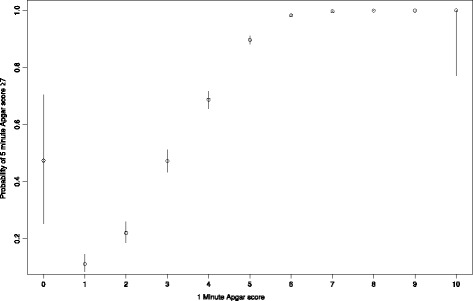
The probability (with 95% CI) of an Apgar score at 5 min (≥7) given any Apgar score at 1 minute

The probability of a 5 min Apgar ≥7 given any 1 min Apgar score is shown in Fig. 2. The wide confidence intervals for 1 min Apgar scores of 0 or 10 are indicative of the small number of births and the variability of neonates receiving those scores. With the exception of the results for a 1 min Apgar score of 0, there is a clear, monotonically increasing effect of greater 1 min Apgar scores being associated with greater probabilities of a 5 min Apgar ≥7.

The descriptive analysis of the relationship between 1 and 5 min Apgar scores in all 267,920 births provides the background against which to consider factors associated with recovery from a 1 min Apgar score <4. Around 0.59% (*n* = 1,580) of all the neonates received a 1 min Apgar score <4. Of those 1,580 neonates, 29.4% (*n* = 465) recovered to a 5 min Apgar ≥7. Table 2 shows a bivariable logistic regression analysis of the association between the predictors and 5 min Apgar recovery.

**Table 2:** Bivariate analysis of recovery from a critically low 1 minute to a normal 5 minute Apgar

		5 minute Apgar				95% CI	
Factor		<7	≥7	Total	Odds ratio	Lower	Upper
Age	20-29	688	274	962	1		
	<20	108	37	145	0.86	0.57	1.27
	30-39	299	138	437	1.16	0.91	1.48
	40	20	16	36	2.01*	1.01	3.93
Ethnicity	Malay	525	344	869	1		
	Chinese	47	23	70	0.75	0.44	1.24
	Indian	39	25	64	0.98	0.58	1.64
	Orang Asal	286	15	301	0.08***	0.04	0.13
	Other	63	15	78	0.36***	0.2	0.63
	Missing	155	43	198	0.42***	0.29	0.60
BMI	Not Obese	800	271	1071	1		
	Obese	303	187	490	1.82***	1.45	2.29
	Missing	12	7	19	1.72	0.64	4.33
Diabetes	Absent	1,057	407	1,464	1		
	Present	58	58	116	2.66***	1.77	3.81
Parity	1	482	252	734	1		
	2-3	439	153	592	0.67***	0.52	0.85
	4-5	137	46	183	0.64*	0.44	0.92
	6	57	14	72	0.47*	0.25	0.84
Hb at delivery	Hb > 11	343	190	533	1		
	Hb ≤ 11	229	65	294	0.51***	0.37	0.71
	Missing	543	210	753	0.7**	0.55	0.89
Baby sex	Female	396	186	582	1		
	Male	584	274	858	1	0.8	1.25
	Missing	135	5	140	0.08***	0.03	0.18
Birth weight	Normal	820	375	1,195	1		
	Low	161	86	247	1.17	0.87	1.56
	Missing	134	4	138	0.07***	0.02	0.16
Foetal distress	Absent	1,014	398	1,412	1		
	Present	101	67	168	1.69**	1.21	2.35
Induced	No	765	348	1,113	1		
	Yes	350	117	467	0.73*	0.57	0.94
Delivery type	Normal	572	144	716	1		
	Breech	19	3	22	0.63	0.15	1.87
	Vacuum Extraction	107	41	148	1.52*	1.00	2.27
	Elective CS	19	30	49	6.27***	3.46	11.64
	Emerg. CS	265	242	507	3.62***	2.82	4.68
	Missing	133	5	138	0.15***	0.05	0.34
Delivered by	Doctor	585	410	995	1		
	Nurse	387	49	436	0.18***	0.13	0.25
	Other^a^	10	1	11	0.14	0.01	0.75
	Missing	133	5	138	0.05***	0.02	0.12

*p < .05, **p < .01, ***p < .001 based on a Wald test

^a^Small cell size < 5

All the factors showed a significant association with recovery from a 1 min Apgar score <4 to a 5 min Apgar ≥7. Missing data were also significantly associated with poorer outcomes in the case of ethnicity, haemoglobin (Hb) at delivery, neonatal sex, birthweight, type of delivery, and the personnel performing the delivery.

Maternal obesity, and the presence of maternal diabetes were both significantly associated with a better chance of Apgar recovery than being normal weight or non-diabetic. Neonates with 1 min Apgar score <4 born to first time mothers were significantly more likely to recover. An Hb > 11 was associated with a significantly greater chance of recovery, as was delivery by vacuum extraction or delivery by Caesarean section (elective or emergency). Induction of labor was associated with poorer odds of Apgar recovery. In contrast foetal distress was associated with significantly better odds of Apgar recovery. Delivery by a doctor was also associated with significantly better odds of Apgar recovery than delivery by any other personnel.

Disentangling some of the associations in a multivariable analysis is complicated by the type of delivery and the personnel conducting the delivery. Who delivers and how they are delivered are strongly correlated. Only doctors performed all the breech deliveries and Caesarean sections, and all but one of the 148 vacuum extractions. Thus, the association between the type of delivery and Apgar recovery is essentially an analysis of doctors’ deliveries. An analysis of the association between the personnel conducting the delivery and Apgar recovery is consequently reduced to an analysis of uncomplicated vaginal deliveries, because these are the only type of delivery that personnel other than doctors also performed. Of the 716 uncomplicated vaginal deliveries, 98.3% were delivered by either a doctor (*n* = 269) or a nurse-midwife (*n* = 435) effectively reducing the comparison to one of doctors and nurse-midwives.

Two independent multivariable logistic regression analyses were performed. The outcome in both cases was Apgar recovery. The first analysis was restricted to the 704 uncomplicated vaginal deliveries by doctors and nurse-midwives and included all the remaining factors from the bivariable analyses. The second analysis was restricted to the 994 deliveries by Doctors with a known type of delivery and included all the other factors from the bivariable analyses. The results are shown in Table 3.

**Table 3:** A mutlivariable analysis of recovery from a critically low 1 minute to a normal 5 minute Apgar score

		Normal vaginal deliveries (*N* = 704)^b^			Deliveries by doctors (*N* = 995)^c^		
			95% CI			95% CI	
Factor		OR	Upper	Lower	OR	Upper	Lower
Age	20-29	1			1		
	<20	1.46	0.74	2.81	1.69	0.99	2.89
	30-39	1.39	0.82	2.34	1.15	0.83	1.59
	40	3.23	0.56	15.72	2.57	1	7
Ethnicity	Malay	1			1		
	Chinese	0.47	0.15	1.26	1.03	0.56	1.89
	Indian	5.13**	1.74	16.46	0.75	0.40	1.37
	Orang Asal	0.12***	0.04	0.29	0.34**	0.17	0.64
	Other	0.22*	0.06	0.65	1.35	0.64	2.83
	Missing	0.67	0.36	1.21	0.82	0.52	1.28
BMI	Not Obese	1			1		
	Obese	1.31	0.83	2.07	1.34*	1.00	1.8
	Missing	5.34^a^	0.27	77.09	0.99	0.35	2.66
Diabetes	Absent	1			1		
	Present	2.09*	1.00	4.5	1.28	0.83	1.98
Parity	1	1			1		
	2-3	0.72	0.44	1.17	0.91	0.66	1.25
	4-5	0.61	0.25	1.38	0.83	0.49	1.40
	6	0.78	0.21	2.48	0.60	0.24	1.43
Hb at delivery	Hb > 11	1			1		
	Hb ≤ 11	0.64	0.32	1.23	0.79	0.52	1.21
	Missing	0.81	0.51	1.28	0.63**	0.47	0.84
Baby sex	Female	1			1		
	Male	1.15	0.75	1.77	0.83	0.63	1.09
	Missing^a^	0.00	–	–	0.00	–	–
Birth weight	Normal	1			1		
	Low	1.07	0.57	1.93	1.16	0.82	1.64
	Missing	1.88^a^	0.09	14.00	1.09	0.13	7.57
Foetal distress	Absent	1			1		
	Present	0.72	0.1	3.2	0.87	0.6	1.26
Induced	No	1			1		
	Yes	0.88	0.53	1.46	1.12	0.81	1.53
Delivery type	Vaginal				1		
	Breech				0.34	0.08	1.07
	Vacuum Extraction				0.79	0.50	1.24
	Elective CS				2.67**	1.39	5.23
	Emerg. CS				1.70**	1.23	2.37
Delivered by	Doctor	1					
	Nurse	0.41***	0.26	0.63			

*p < .05, **p < .01, ***p < .001 based on a Wald test

^a^Small cell size < 5

^b^Estimates adjusted for all other variables in the table except “Delivered by”

^c^Estimates adjusted for all other variables in the table except “Delivery type”

Separate sub-analyses were conducted for vaginal deliveries only comparing Doctors and Nurses (*n* = 704), and Doctor deliveries only comparing the type of delivery (*n* = 995)

In uncomplicated vaginal deliveries, after controlling for other factors, the chance of Apgar recovery was significantly worse when the delivery was performed by a nurse-midwife than by a doctor. A neonate with a 1 min Apgar score <4 with an uncomplicated vaginal delivery by a doctor had odds of Apgar recovery 2.4 times greater than a neonate with a 1 min Apgar score <4 delivered by a nurse-midwife (95% CI: 1.59–3.85). Poorer recovery was independently associated with being a neonate with a 1 min Apgar score <4 born to an Orang Asal or “other” ethnicity mother compared with being born to a Malay mother. In contrast, neonates with 1 min Apgar scores <4 born to Indian mothers were more likely to recover. Being a neonate with a 1 min Apgar score <4 born to a diabetic mother was also associated with better outcomes (OR = 2.09, 95% CI:1–4.5).

In deliveries performed exclusively by doctors, after controlling for other factors, Apgar recovery was more likely following a Caesarean section (emergency or elective) than it was following an uncomplicated vaginal delivery. A neonate with 1 min Apgar score <4 delivered by elective CS had odds of Apgar recovery 2.7 times greater than a neonate with a 1 min Apgar score <4 delivered by uncomplicated vaginal delivery (95% CI: 1.39–5.23); and a neonate with a 1 min Apgar score <4 delivered by emergency CS had odds of Apgar recovery 1.7 times greater than a neonate with 1 min Apgar score <4 delivered by uncomplicated vaginal delivery (95% CI: 1.23–2.37). Recovery was not significantly different following vacuum extraction. Independent of other factors, neonates with 1 min Apgar scores <4 born to obese mothers were more likely to recover than those neonates with 1 min Apgar scores <4 born to non-obese mothers. Poorer recovery, compared with neonates with 1 min Apgar scores <4 born to Malay mothers was also independently associated with being a neonate with a 1 min Apgar score <4 born to an Orang Asal mother.

## Discussion

Approximately 0.59% of live term births had 1 min Apgar score <4, of which only 29.4% recovered to a 5 min Apgar ≥7. The 1 min Apgar score is indicative of how well the newly born manages the immediate transition from intrauterine to extrauterine life [6]. Previous studies have examined factors associated with 1 min Apgar scores <7, and found significant associations with, among other factors, prematurity, postmaturity, low birthweight, and breech delivery [19–21]. This study was quite different because of the focus on recovery of term neonates with 1 min Apgar score <4 – and excluded in the sampling strategy prematurity, postmaturity, and congenital anomalies. As we consider our findings in relation to the earlier literature, it should be borne in mind that our focus on live term births may explain any differences.

### Delivery type and personnel factors

Berglund and colleagues found that the 1 min Apgar score was associated with the manner in which the labour itself was managed by health care staff [22]. That is, in addition to any pre-existing risk factors such as birth weight or gestational age, the clinical decisions made during labour and the practice of the labour ward had an effect on that immediate transition to extrauterine life. A recent systematic review of deliveries in the US found no significant difference in the prevalence of low Apgar scores in deliveries by doctors or by nurse-midwives [23]. In our study, among the uncomplicated vaginal deliveries there was a clear, independent association between the qualification of the person conducting the delivery (doctor or nurse-midwife) and Apgar recovery. Neonates with 1 min Apgar scores <4, delivered by nurse-midwives had odds of recovery 40% of those babies delivered by doctors (95%CI:.26–.63).

The earlier findings and these findings, however, should not be regarded as contradictory. The probability of a poor 1 min Apgar score could be identical for doctors and nurse-midwives, and the differences in outcome may have a number of explanations. First, given a low Apgar score doctors may simply be better equipped to manage the recovery. This may in turn lead to speculation about whether nurse-midwives require additional training in resuscitative techniques; [24] and/or whether structurally the health system needs to make changes to manage neonates with 1 min Apgar scores <4 [22] The second explanation, is that when nurse-midwives anticipate a poor outcome they are more likely to refer it to a doctor; and because the poor outcome was anticipated, there is time to prepare an appropriate clinical response. In contrast, the un-anticipated poor outcome will not be referred, and there will not be the same time to prepare an appropriate clinical response. This is a form of selection bias in which nurse-midwife’s have to manage a more complicated clinical situation than doctors.

The evidence on the association between the type of delivery and birth outcome is mixed, and highly dependent on the presenting clinical features at the time of delivery. Two systematic reviews (2006 and 2012) comparing planned caesarean delivery versus planned vaginal birth, for instance, identified no studies of sufficient quality quality to inform a scientific view [25, 26]. Whether a CS is planned or unplanned also introduces complicating factors such as the timing, and the medical reasons underpinning the decision [26].

Given a 1 min Apgar score <4, however, there was an independent association between the type of delivery and recovery. Specifically, neonates with 1 min Apgar scores <4 delivered by CS (emergency or elective) had significantly better odds of recovery than neonates with 1 min Apgar scores <4 delivered by uncomplicated vaginal deliveries. Elective CS was associated with odds of recovery 2.7 times greater than uncomplicated vaginal deliveries (95% CI: 1.39–5.23); and emergency CS was associated with 1.7 times greater odds of recovery (95% CI: 1.23–2.37). This may be an effect of the degree of trauma associated with different types of delivery. If CS births do result in less birth trauma in this cohort, then it would make sense that they would recover faster. The data may also point to issues in the identification of births requiring CS. Around half of the deliveries performed by doctors, that involved 1 min Apgar scores <4, did not receive an emergency CS. It is also worth noting that the hospitals’ protocol is for spinal anaesthesia in CS, so the change in Apgar is unlikely to reflect post-anesthesia recovery.

The findings on delivery personnel and type of delivery become useful hypothesis generating mechanisms for possible future research. While not definitive, the findings suggest that a planned investigation of labour ward practice for those relatively rare, 1 min Apgar scores <4 could help to identify strategies that would improve recovery rates.

### Maternal clinical factors

In the unadjusted analyses, we found that the odds of recovery were better in neonates with 1 min Apgar scores <4 born to mothers with diabetes and mothers who were obese. We also found that foetal distress was associated with better odds of recovery, and that the odds of recovery were significantly worse when the mother had low Hb (<11). Most of these associations disappeared in adjusted analyses. BMI and diabetes were the exceptions.

A number of recent studies have reported a negative association between maternal BMI and birth outcomes, including Apgar score [27–30]. At least one recent study, however, found no significant association [31]. We found no association between BMI and the odds of recovery in the analysis of uncomplicated vaginal deliveries. In contrast to all the results showing a negative or neutral association between maternal BMI and birth outcomes, in the analysis of deliveries by doctors, we found that neonates with 1 min Apgar scores <4 born to obese mothers had a small, but significantly better chances of recovery than those born with 1 min Apgar scores <4 to normal weight mothers (OR = 1.34; 95% CI:1.00–1.8).

The literature on maternal diabetes and low Apgar score is not clear cut, but tends towards worse outcomes; [32, 33] although in one recent study that found worse Apgar scores associated with maternal diabetes, the association disappeared in an adjusted analysis [34] Whether the outcome was worse also appears to be associated with the level of glycaemic control [35]. Again, and in contrast with the results on birth outcome, the odds of recovery given a low 1 min Apgar score were better in uncomplicated vaginal deliveries born to mothers with diabetes.

The differences in the associations between the birth outcome data and the Apgar recovery data are noteworthy and raise rather than answer questions. It may be, for instance, that mothers with known diabetes or high BMI trigger a hyper-vigilant clinical care response. A neonate’s initial Apgar score may be <4, but because of the preparedness of the staff for a poorer outcome they may also be better prepared to respond.

### Ethnicity

Numerous studies have reported ethnic variations in birth outcomes [36, 37]. Some of the variation appears to be attributable to biology, [38, 39] but there is also substantial evidence for social and economic factors driving differences, [36, 40] and not always in the direction of minority groups being worse off [41, 42]. The results necessarily raise questions about the differential and synergistic effects of genetics, culture, and environment [37, 43]. In the present study, in the adjusted analysis for uncomplicated vaginal deliveries, Indian neonates with 1 min Apgar scores <4 (a minority group) had odds of recovery 5 times greater than Malay neonates with 1 min Apgar scores <4 (the majority group) (OR:5.13; 95% CI: 1.74–16.46). In contrast, Orang Asal and “Others” had substantially lower odds of recovery than Malay neonates with 1 min Apgar scores <4 (OR:.12; 95% CI: .04–.29 and OR:.22; 95% CI: .06–.65 respectively). In deliveries by doctors, only the Orang Asal neonates with 1 min Apgar scores <4 were significantly different (worse off) from the Malay neonates with 1 min Apgar scores <4 (OR:.34; 95% CI: .17–.64).

One possible explanation for the worse outcomes for the Orang Asal lie in their comparatively more geographically isolated living conditions, which may give rise to fewer antenatal visits and a reduced opportunity to provide on-going obstetric care and risk assessment. Orang Asal mothers may also be physically less healthy during their pregnancy [44]. Finally, there may be health systems issues, including accessibility, leading to poorer healthcare for indigenous populations; this notwithstanding Malaysia’s historically strong performance in improving maternal and child health outcomes [45].

Data for 2012 from the Malaysian government’s Economic Planning Unit showed the “Other” ethnic group to have the lowest mean monthly income; [46] and Orang Asal are over represented in the “poverty” and “hardcore poverty” statistics [44]. This would also suggest socioeconomic drivers, but given the universal coverage of maternal and child health services in Malaysia, wealth/poverty may not be a complete explanation for the results.

### Missing data

The problem of collecting high quality labour ward data is not new [47, 48]. Where missing data are usually treated as a problem to be overcome, [49] missing data can also be treated as informative [50]. Rather than using data imputation to fill in the blanks, [51] in this study we elected to model the missing category explicitly.

In the unadjusted models missing data on ethnicity, Hb, the neonate’s sex, birth weight, type of delivery, and the person conducting the delivery were all associated with lower odds of recovery than the base category. In the adjusted models, no category of “missing” was significantly different from the respective base categories, and in a number of cases the cell sizes were so small that estimating confidence intervals was impossible.

Nonetheless, the existence of the missing data does hint at something interesting about the relationship between the urgency of neonatal clinical need and data quality. It is conceivable that when a neonate is critically ill, recording the sex or birth weight is seen as less relevant, or more concerning it may point to a deeper issue of quality care.

### Limitations

There are important limitations associated with the use of registry data [48]. For instance, a small number of births (*n* = 21) with Apgar scores of 0 at 1 and 5 min were excluded as stillbirths, because they likely were [21]. There is the possibility of a 0/0 Apgar score followed by successful resucitation [52]. The manner of data collection in registries, however, often relies on simplifying assumptions and these need to be understood. In spite of the limitations, registry data can make important contributions to quality improvement, clinical research, and policy development [53].

In this section, three points are discussed: coverage, residual confounding, and the reliability of the outcome measures (Apgar score).

The completeness and coverage of the registry is important for the population to which the data speak [54]. In the case of the NOR, the data were drawn comprehensively from the 14 major hospitals which account for around 27% of births nationally. One might be reasonably comfortable generalising the findings to those hospitals with requisite additional caution in drawing wider conclusions. In hospitals not represented in the registry, which includes Government district level hospitals and private hospitals, the number and qualification-mix (i.e., doctors and nurse-midwives) of staff, their training, and the equipment may vary. All of these could affect outcomes, and therefore generalisability.

The granularity of the data from general administrative registries is necessarily going to be lower than they would be in cohort studies looking at specific questions. Choices need to be made about the limited kinds of data that can be collected routinely within a functioning health care unit that is not dedicated to research. In choosing to record certain data and not other data, there is an obvious concern with residual confounding; [54] that is, failing to account for a relevant factor in the adjusted analyses. Apgar recovery is likely, for example, to be strongly associated with the resuscitative skills, technology, and protocols available on each of the labour wards across the 14 hospitals. This is not recorded in the registry, but critical for drawing more definitive conclusions from the data. Notwithstanding the issues of residual confounding, as part of a more general study of possible associations, these kinds of data fulfil an important hypothesis generating role, including hypotheses about other possible unmeasured factors.

Finally, there is some question about the capacity of healthcare professionals to make valid and reliable assessments of neonatal Apgar scores [55–57]. One of the studies that highlighted issues of reliability in Apgar assessment was based on the evaluation of neonates from 23 to 40 weeks gestation, [58] and the other considered very low birth weight neonates with a range of gestational ages [56]. The design of the reliability studies ignored the very high base rate of Apgar scores ≥7 and selected a wider range and more critical clinical presentations than would be expected on a normal delivery ward. In this study all the neonates with 1 min Apgar scores <4 had reached term, and one might anticipate that term singleton births are easier to assess with greater reliability. Futhermore, even allowing for variation in staff clinical assessment, the vast majority of the neonates had Apgar scores ≥7: 97.25% of neonates had a 1 min Apgar score ≥7 and 99.3% had a 5 min score ≥7. Accepting the average variation in Apgar assessment across clinical staff of 2.4 points, the separation used in this study between a 1 min Apgar scores <4 and an 1 min Apgar score ≥7 ensured little room for error in category.

The results of this study speak most directly to the 14 state tertiary hospital contributing data to the NOR – around 27% of national births. They may arguably extend to other government hospitals which operate under similar policies and practice guidelines (a further 58% of national births); however, those hospitals will also have different levels of specialisation. It seems less likely that the results would generalise to private hospitals (15% of national births) which would operate under their own policies and guidelines.

## Conclusions

This study is the first empirical investigation of factors predicting the transition from the first minute to the fifth minute of life in babies with 1 min Apgar scores <4. As such the findings are best seen in the role of raising questions and generating hypotheses. The vast majority of births have Apgar scores above the threshold of 3 (99.3%), and the majority of those are successfully delivered by nurse-midwives. In the relatively rare event of a 1 min Apgar score <4, only 29% of neonates recover to an Apgar score ≥7 by the fifth minute.

After controlling for other factors, the qualification of the person performing the delivery and the type of delivery are significantly associated with recovery in those few neonates with Apgar scores <4. Do the differences arise because of the cases (a selection bias), the management, or the practitioners’ skills? These questions all point to possible health system explanations.

By their administrative nature, however, obstetrics registries tend not to collect the kind of detailed data that could be used to disentangle the specific events. Nonetheless by noting statistical differences in outcomes, the results provide a jumping off point for further investigations which would need an audit of the specific labour room practices for neonates with 1 min Apgar scores <4.

## Abbreviations

BMI: Body mass index
CS: Caesarean section
GLM: Generalized linear modeling
Hb: Haemoglobin
NOR: National Obstetrics Registry
OR: Odds ratio
